# Supplementary figures and images for: Energy Drink Consumption among Adolescents Attending Schools in Lubumbashi, Democratic Republic of Congo
Source: Int J Environ Res Public Health. 2021 Jul 17;18(14):7617. doi: 10.3390/ijerph18147617 (PMC8304143; doi:10.3390/ijerph18147617)

Figure S1

*Bora Boom*

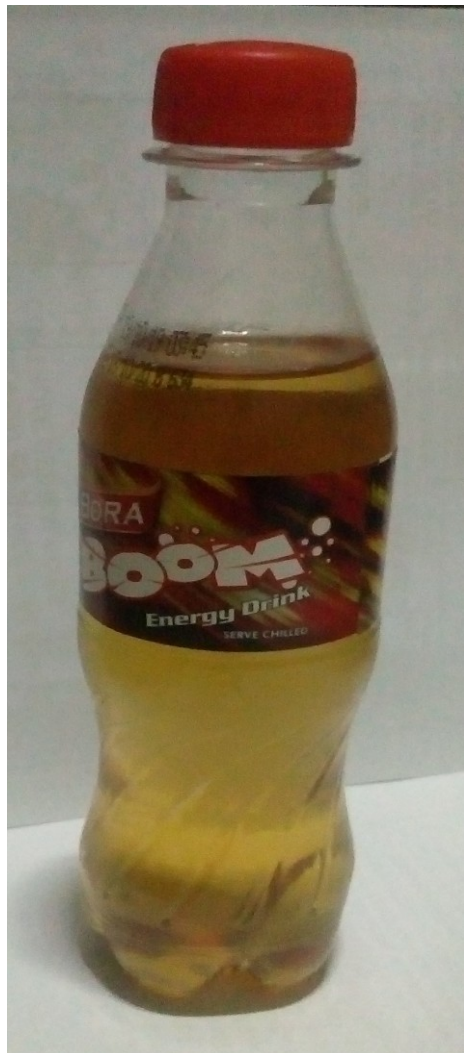

*Kung Fu*

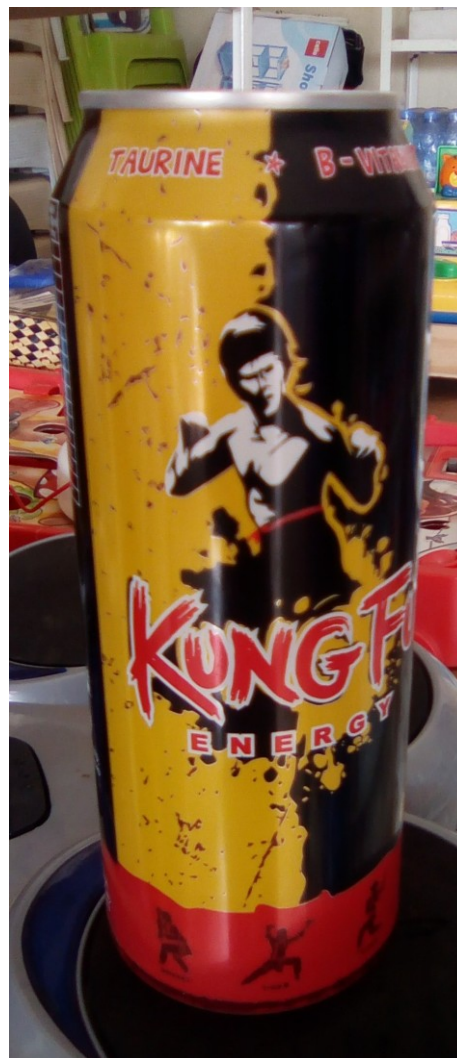

*XXL*

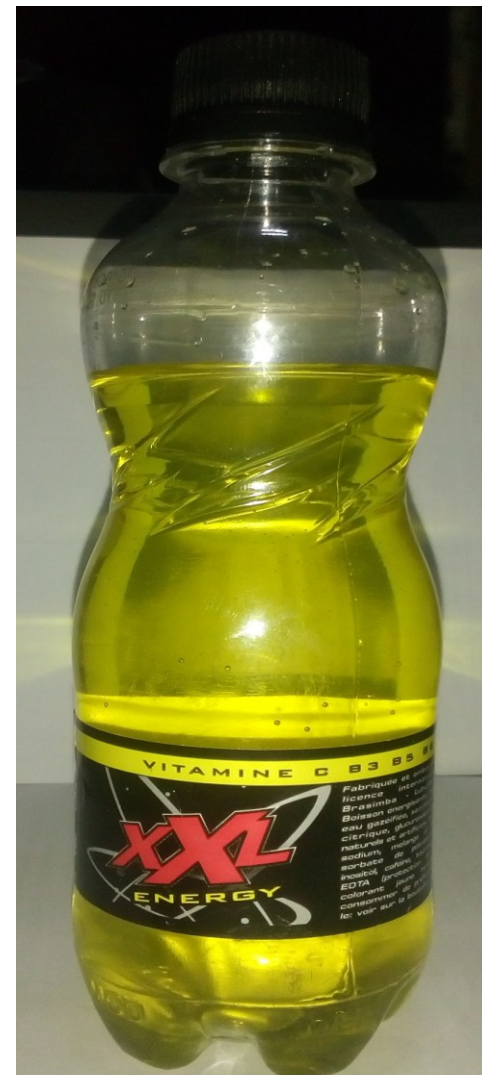

Figure S2

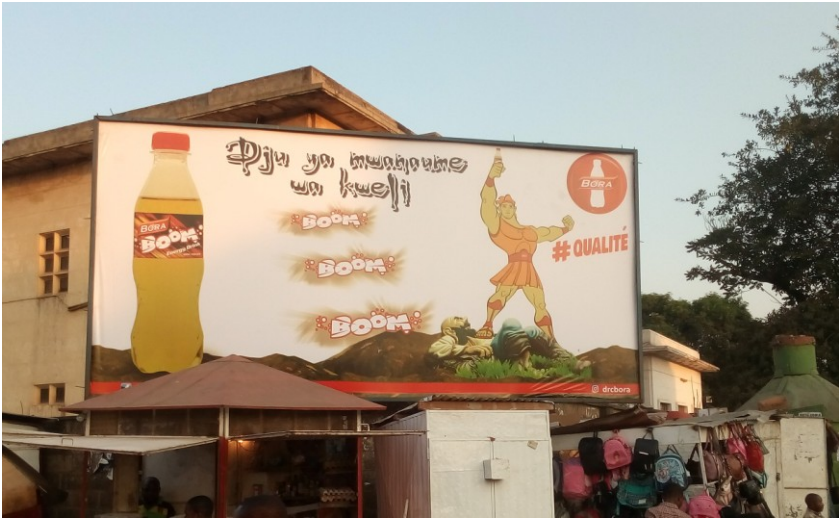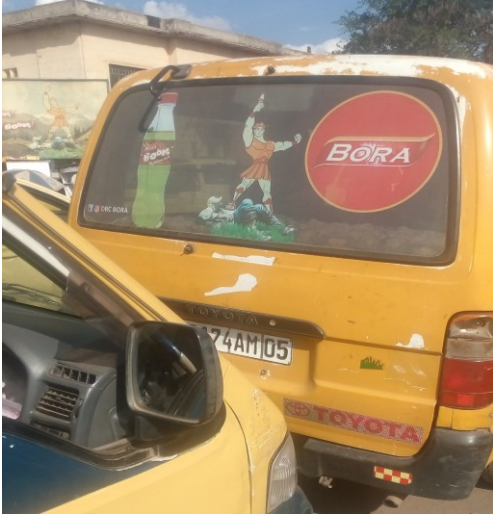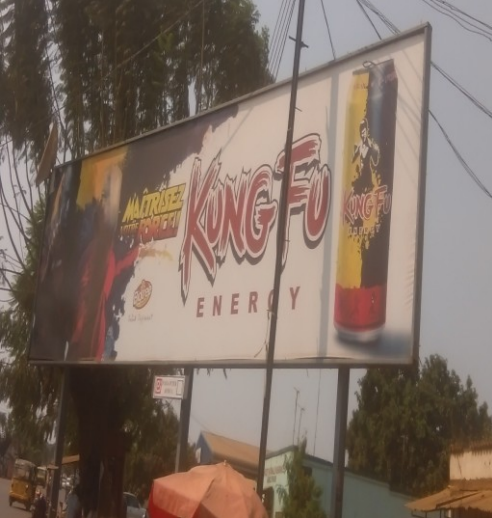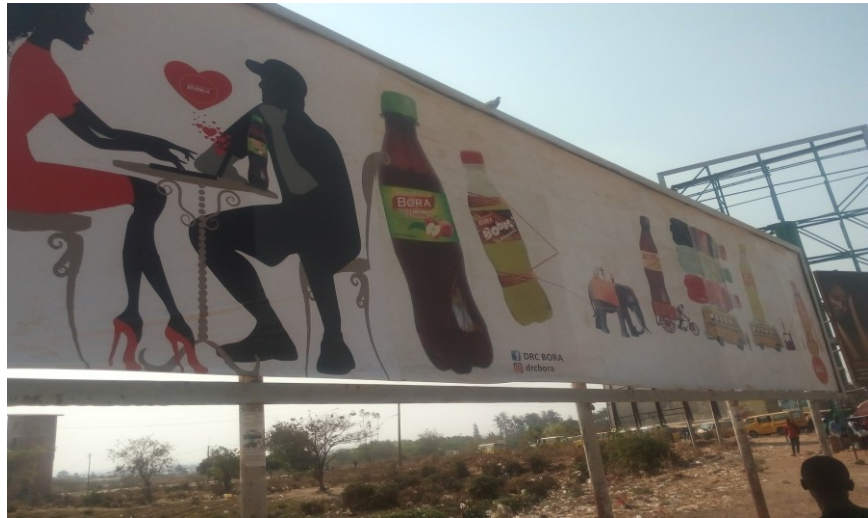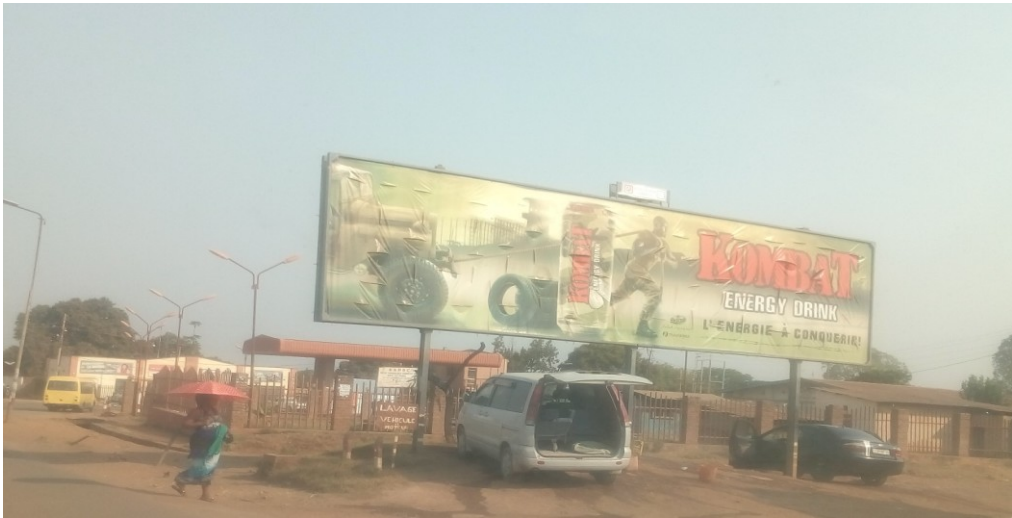

Figure S3

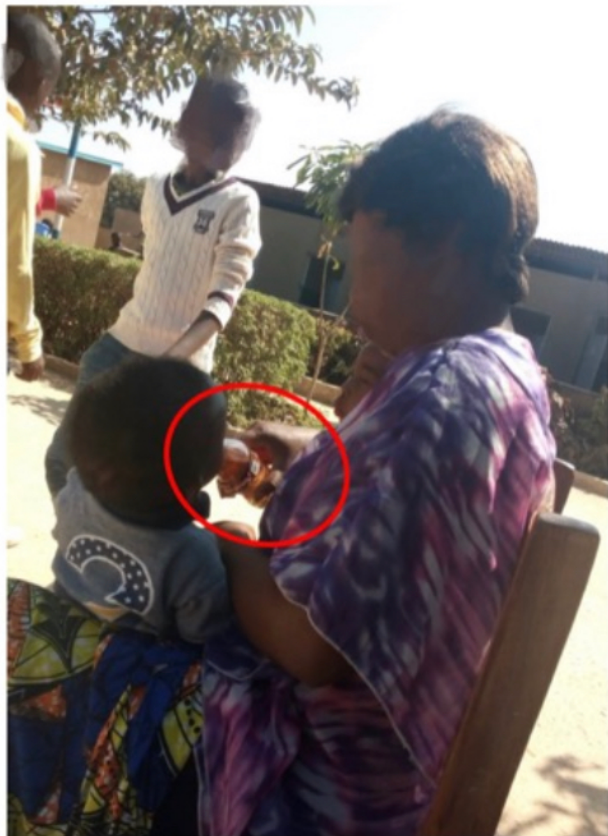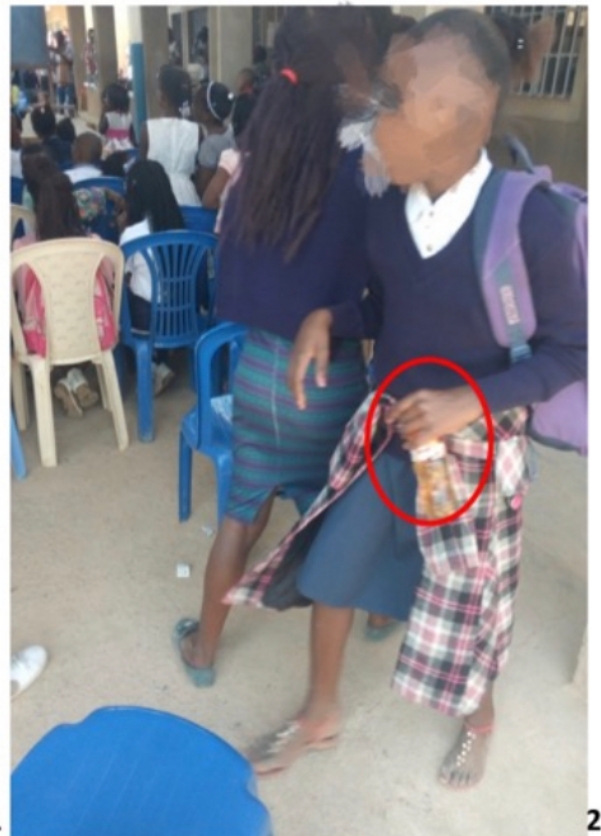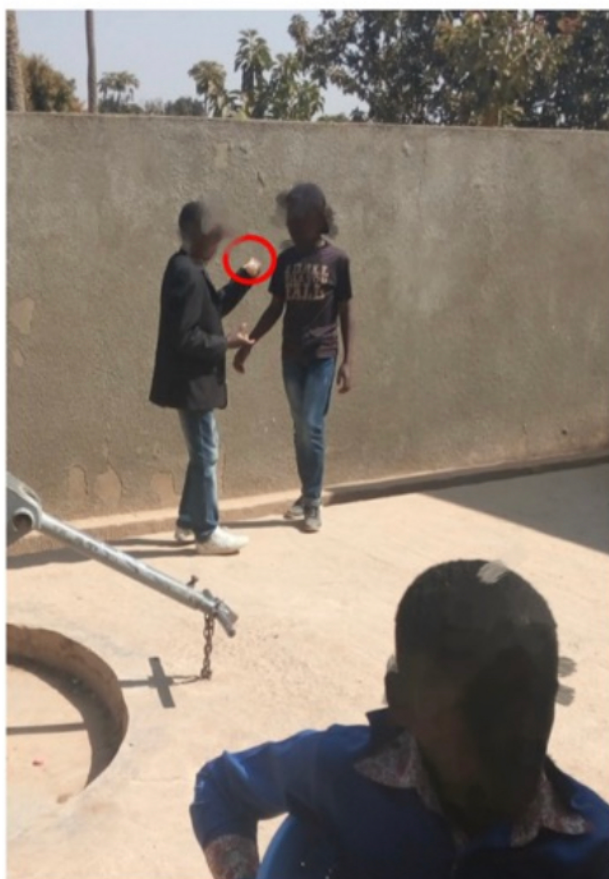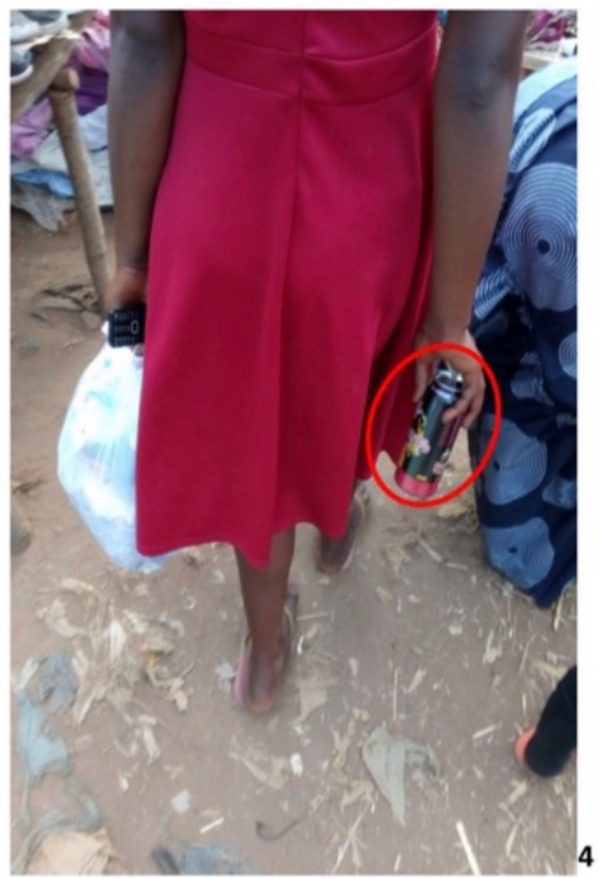

Supplement: Supplementary file 1 [file ijerph-18-07617-s001.zip › ijerph-1265830-supplementary.pdf]
